# Identifying large-scale interaction atlases using probabilistic graphs and external knowledge

**DOI:** 10.1017/cts.2022.18

**Published:** 2022-02-11

**Authors:** Sree K. Chanumolu, Hasan H. Otu

**Affiliations:** Department of Electrical and Computer Engineering, University of Nebraska-Lincoln, Lincoln, Nebraska, USA

**Keywords:** Interactome, atlas, gene interaction network, external knowledge, Bayesian networks

## Abstract

**Introduction:**

Reconstruction of gene interaction networks from experimental data provides a deep understanding of the underlying biological mechanisms. The noisy nature of the data and the large size of the network make this a very challenging task. Complex approaches handle the stochastic nature of the data but can only do this for small networks; simpler, linear models generate large networks but with less reliability.

**Methods:**

We propose a divide-and-conquer approach using probabilistic graph representations and external knowledge. We cluster the experimental data and learn an interaction network for each cluster, which are merged using the interaction network for the representative genes selected for each cluster.

**Results:**

We generated an interaction atlas for 337 human pathways yielding a network of 11,454 genes with 17,777 edges. Simulated gene expression data from this atlas formed the basis for reconstruction. Based on the area under the curve of the precision-recall curve, the proposed approach outperformed the baseline (random classifier) by ∼15-fold and conventional methods by ∼5–17-fold. The performance of the proposed workflow is significantly linked to the accuracy of the clustering step that tries to identify the modularity of the underlying biological mechanisms.

**Conclusions:**

We provide an interaction atlas generation workflow optimizing the algorithm/parameter selection. The proposed approach integrates external knowledge in the reconstruction of the interactome using probabilistic graphs. Network characterization and understanding long-range effects in interaction atlases provide means for comparative analysis with implications in biomarker discovery and therapeutic approaches. The proposed workflow is freely available at http://otulab.unl.edu/atlas.

## Introduction

Individual elements of a biological system work in concert at the molecular level, which is best analyzed and explained within the context of networks. Networks involving all direct and indirect interactions between genes and/or gene products (the interactome) can be used to understand biological pathways and disease mechanisms. Such an understanding and tools for in silico manipulation lead to new innovative, noninvasive, cost-effective, and scalable approaches to combat human disease by providing means to manipulate and model molecular mechanisms in an efficient and effective way [1].

Conventional methods for interaction network construction used correlation [2-5] or mutual information [6-9]-based measures. Although dependent- or coexpression may lead to functional similarity [10,11], these approaches produced bulky networks and were based on pairwise associations only [12-15] ignoring higher-level associations that may not be inferred by strong individual, paired associations. More complex methods emerged using Bayesian networks (BN) [16-23], Gaussian graphical models – simultaneous equation models [24-26], state space models [27-29], machine learning [30,31], and statistical methods [32,33]. These methods overcome the limited view of the pairwise approaches that are generally based on linear associations by providing a probabilistic blanket of dependency and coregulation, modeling nonlinear associations. However, they can learn networks for only a limited number of nodes [34] because of their high computational complexity. Indeed, biological systems operate at scales much larger than the network sizes handled by these approaches [35,36], which must resort to dissecting the system into pathways. To perform true system-level analysis, there is a need for tools that infer interaction networks, or interaction atlases, at levels beyond the current pathway views.

While the ever-increasing biological data production in the fields of genomics, transcriptomics, and proteomics has resulted in a plethora of approaches attempting to recover interaction networks in biological systems [37,38], they have not always made efficient use of the vast amount of annotated data available [39]. This valuable resource can be used in a systematic way to guide methods that learn interaction networks [40]. Traditional methods for gene interaction (GI) network construction use linear measures and generate large interactomes that miss nonlinear relationships and ignore external knowledge [4,8,41,42]. Another group of methods either uses a single external knowledge source or requires the exact gold-standard network for the one to be generated [43-45], which are impractical approaches in real settings. A third group of approaches focuses on a limited view of interactions (e.g., finding regulatory elements only, or identifying functional associations as interactions) or uses deterministic mechanisms for external knowledge incorporation [46-48]. These last two groups of methods perform well only for a few hundred to one- or two-thousand genes due to algorithm complexity.

In this paper, we propose a method that uses a diverse set of knowledge bases to infer interaction between two genes based on a stochastic, automated framework. Our approach fuses this information with experimental data in a probabilistic graph representation to generate a large-scale interactome (a few tens of thousands of genes). The proposed approach provides a higher system-level view to understand the biological mechanisms in health and disease. We see limited efforts in this direction using linear models or models that do not incorporate external knowledge in building large-scale networks [49,50]. Although these are helpful, there is a need for advanced computational methods that make use of the existing interaction knowledge that is external to the experimental data.

The algorithm described in this paper uses a novel divide-and-conquer approach to construct interaction atlases. Instead of learning the entire large interaction atlas in one shot, we first divide the nodes into groups based on their expression profiles. The interaction network within each cluster is learned using both experimental data and external knowledge. One representative gene in each cluster is selected to build the network between the cluster representatives. This forms a “network of clusters” and is used to merge clusters that are linked together by building an interaction network using the union of the nodes in the two clusters. The ensemble of the links after the “merge” process yields the final atlas.

The proposed method is distinct from existing approaches that dissect biological networks, such as the module network representation [51], which constrains the nodes in a *module* to having the same parents or tree-based methods [52], which model recovery as a feature selection problem based on ranked lists obtained from regression analysis or the stochastic block model-based approaches where families of distributions are defined for the nodes, resulting in unscalable node classification and parameter estimation problems [53,54]. We establish the proposed method using optimized clustering, structure learning, external knowledge incorporation, representative gene selection, and cluster merging processes. The utility of our approach is demonstrated with simulated data and compared to correlation and information theory-based approaches that are used for large-scale interaction atlas generation.

## Materials and Methods

**Fig. 1.**
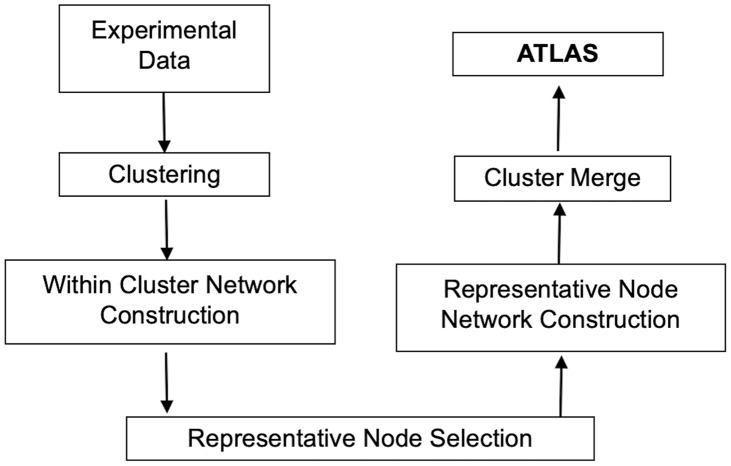
Workflow for atlas generation.

The proposed atlas generation method is shown in Fig. 1. In what follows, we describe each module in Fig. 1 in detail.

### Experimental Data

To obtain simulated data that follow an interaction network representing true mechanisms, we considered the human pathways found in the Kyoto Encyclopedia of Genes and Genomes (KEGG) pathway database [55]. KEGG pathways involve gene products, compounds, maps (a.k.a. pathways), DNA, RNA, and other molecules. In total, we obtained 337 human pathways, and for each pathway, we deduced all of the direct and indirect gene-GIs using KEGG2Net [56]. We analyzed the KGML files for these pathways to extract the map (pathway) entries that the genes are directly or indirectly linked to. We merged the KEGG2Net GI networks obtained for each pathway following the direct/indirect links between the gene and map entries in the KEGG pathways. For example, let the set G_XY_ represent all of the genes in pathway X that has a direct/indirect link to the map (pathway) Y represented as a “node” in pathway X. Similarly, let the set of genes G_YX_ represent all of the genes in pathway Y that has a direct/indirect link to the map (pathway) X represented as a “node” in pathway Y. Then, we establish a link between elements of the gene sets G_XY_ and G_YX_. By applying this merge method, we obtained an interaction atlas that represents all of the direct and indirect interactions between genes represented by all of the human KEGG pathways, which consisted of 11,454 nodes and 17,777 edges. We used SynTRen v 1.2 [57] to generate simulated transcriptomic data for 20 test and 20 control samples for all of the 11,454 genes represented in our interaction atlas.

### Clustering

We clustered the gene expression data matrix using hierarchical clustering, k-means, clusternomics – an integrative context-dependent clustering for biomedical datasets [58], EMMIXgene – a mixture model-based approach to cluster microarray expression data [59], gama – a genetic approach to maximize a clustering criteria [60], DIANA – a divisive, not agglomerative, hierarchical clustering, FANNY – a fuzzy clustering approach, and PAM – partitioning around medoids [61]. The implementations were done in R v 3.6.3 using the packages (functions) stats (hclust, cutree, k-means) v 4.2.0, clusternomics v 0.1.1, EMMIXgene v 0.1.3, gama v 1.0.3, cluster (diana, fanny, pam) v 2.1.2. Validation of the clustering results was performed using the biological homogeneity index (BHI) [62], V-measure [63], and adjusted Rand index (ARI) [64]. These metrics were calculated using R packages clValid v 0.7, saber v 0.3.2, and mclust v 5.4.7, respectively.

### Incorporation of External Knowledge

As the number of genes in a cluster is expected to be small (tens to a few hundreds), we learned the networks within a cluster using our previously established tool, Bayesian network prior (BNP) [19]. BNP is a construct that learns an interaction network based on external knowledge and experimental data. As part of this paper, we updated our BNP software by updating the evidence matrix BNP uses to infer interaction of two genes. We gathered information from Gene Expression Omnibus [65], KEGG [55], NCI/Nature Pathway Interaction Database [66], Reactome [67], Biological General Repository for Interaction Datasets [68], FunCoup [69], Hetionet [70], HumanNet [71], RegNetwork [72], STRING [73], and GeneMANIA [74] data sources that imply an interaction between two genes based on different evidence types, for example, Affinity Capture assays, colocalization, two-hybrid experiments, and coexpression.

We represented the interaction information in the form of an “evidence matrix” where the columns were the evidence types, and the rows were the pairs of genes. If a pair of genes was labeled as interacting by a data source based on an evidence type, we placed a “1” in that location, which was otherwise left as a “0.” When all the data sources were combined, we obtained an evidence matrix that contained interaction information for 15,725,553 unique pairs of genes. We added a “GI” column to this evidence matrix, and if a pair of genes had two or more evidence types based on which they were known to be interacting, we labeled the GI entry for that pair as “1” and otherwise labeled it as “0.”

BNP is a BN representing the dependency structure between “different experimental evidence types that imply GI” and the “event, GI.” Using our evidence matrix, we learned BNP with the bnlearn R package v 4.6.1 [75] based on the hill climbing structure learning approach [76] utilizing the Bayesian information criterion score [77]. The consensus network was obtained based on 1000 bootstrapped datasets where model averaging was used to calculate the strength of links between the nodes of BNP. The final BNP graph was obtained by only retaining the edges that have significant strength values [78]. Therefore, BNP is itself a BN with one node representing “GI” and the remaining nodes representing “different evidence types.” BNP reflects the distilled representation of acquired scientific knowledge and can be used to calculate the probability of interaction between two genes using a fusion of external and experimental data. The updated version of the BNP used for this paper can be found at http://otulab.unl.edu/BNP.

### Within Cluster Network Construction

Given an expression dataset, the interaction network for the genes in a cluster was calculated using BNP as previously described [19]. BNP uses experimental designs with two groups of samples (e.g., cancer vs. normal) where the set of observations for structure learning is obtained by pairwise comparison of samples in the two groups. As detailed previously [79], this preprocessing step of the expression profiles provides a distribution of expression fold change between the two groups for each node (gene) in the network and has proven to be a reliable and robust way to obtain input data for network learning.

Given two genes, BNP is instantiated with their expression profiles to obtain the value of its GI node that represents their interaction probability based on external knowledge and the supplied experimental data. This probability is calculated for each pair in a set of genes for which an interaction network is to be constructed and incorporated in the structure learning phase to calculate the probability of the candidate graphs. This way the optimum “maximum *a posteriori*” measure is maximized instead of the suboptimum “maximum likelihood” parameter, optimizing the search process as BNP allows for calculation of P(G), the probability of the candidate graph in the search. In the end, the networks learned by BNP represent the GI dynamics for the case under investigation (e.g., cancer) that is used to obtain the experimental data.

### Representative Node Selection

We analyzed the GI networks generated for each cluster by BNP using the central informative nodes in network analysis (CINNA) R package v 1.1.54 [80]. Given a network topology, CINNA first identifies the appropriate centrality measures (out of ∼50 such measures) for the input network. Next, dimension reduction techniques are used to identify the most informative centrality measure. For each network generated by BNP, we applied CINNA to identify the most informative centrality measure for the network and then used that measure to identify the most central node in the network. We chose these nodes as the representative nodes for their clusters.

### Cluster Merge

We sifted the expression values for the representative nodes (genes) of each cluster and used BNP to learn an interaction network for these nodes. Representing each cluster with a gene enabled us to use BNP to build the interaction network of the representative genes. As BNP uses external knowledge to build an interaction network, using a hypothetical gene, for example, the average of all genes in a cluster, or an eigengene in a cluster, would strip BNP off of this feature. The interaction network of representative genes is regarded as a “network of clusters” as each node (gene) represents a cluster. We merged clusters that are linked together by building an interaction network using the union of the genes in the two clusters. The ensemble of the links after the “merge” process yielded the final atlas.

## Results

### BNP Construction

We assessed the validity of our BNP construction using fivefold cross-validation on the GI inference. At each iteration, we left out 20% of the gene pairs from our evidence matrix and learned BNP as described with the remaining 80% of the data. For the left-out pairs, we instantiated BNP using their evidence vector and inferred the GI node as a probability value. We were able to predict the GI node’s state with an area under the curve (AUC) value of 94%. The final BNP model was developed using all of the gene pairs in the evidence matrix as described. We tested BNP on 167 KEGG human pathways that have less than 40 nodes to obtain networks with reasonable complexity. For each pathway, the corresponding GI network and simulated dataset were obtained using KEGG2Net and SynTRen, respectively. The networks learned with BNP and the corresponding conventional structure learning approach that does not use any external knowledge were compared with the true networks. The results, summarized in the Supplementary Data, show that BNP on average attained a 95.52% AUC whereas the structure learning approach that did not utilize external knowledge attained an average AUC of 67.09%. These results demonstrated confidence in the construction and application of BNP to learning GI networks from experimental data using external knowledge. The updated implementation of BNP is freely available at http://otulab.unl.edu/BNP.

### Clustering

**Fig. 2.**
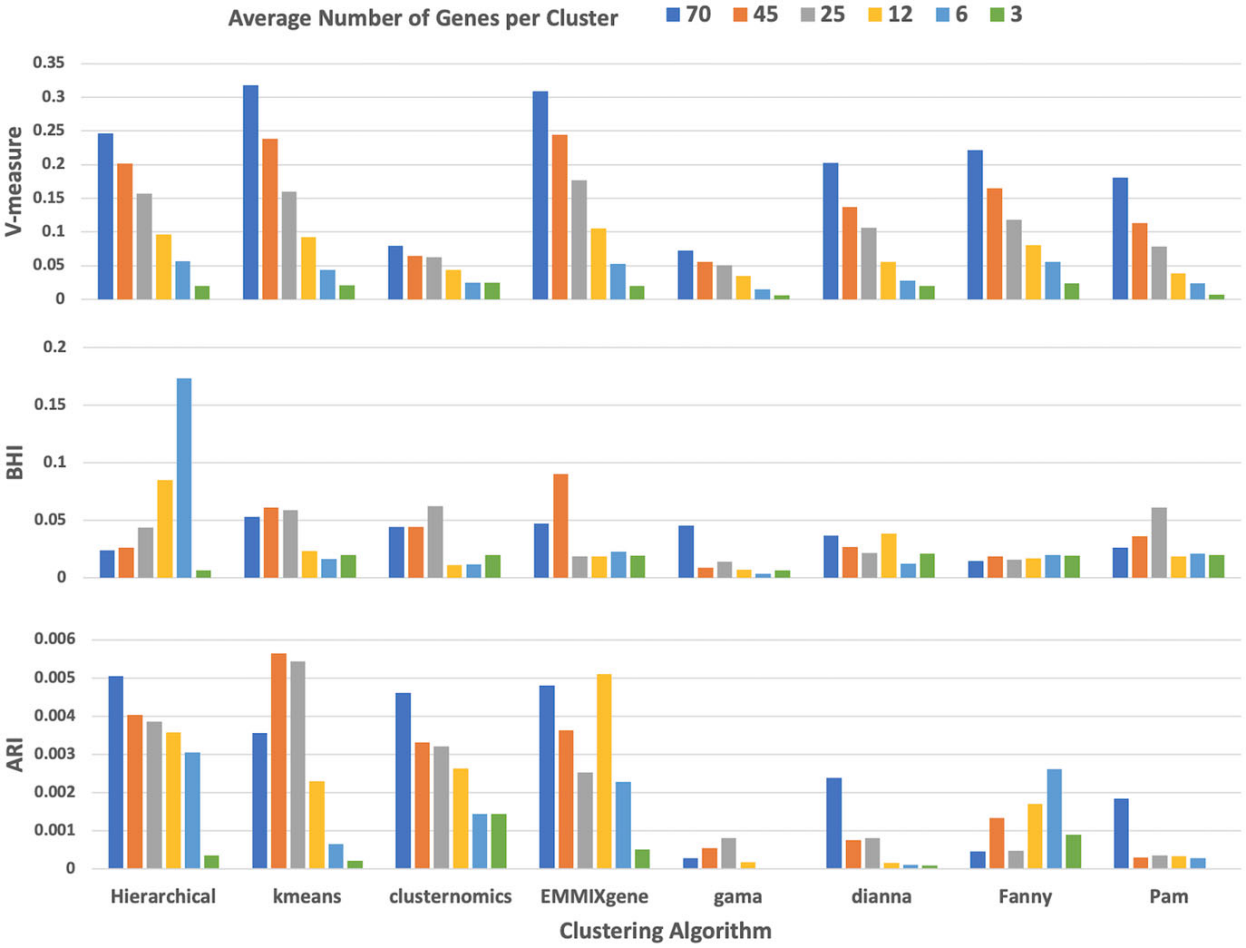
V-measure, biological homogeneity index (BHI), and adjusted Rand index (ARI) values for the eight clustering algorithms using a range of average number of genes per cluster. The input data are the simulated gene expression data that is generated from the human atlas with 11,454 genes representing 337 Kyoto Encyclopedia of Genes and Genomes (KEGG) pathways.

For each of the eight clustering algorithms, we had six runs where we used the following expected number of genes in a cluster: 3, 6, 12, 25, 45, 70. We used the default values for all other hyperparameters required by the algorithms. Our goal was to sample the neighborhood of the average number of genes in a cluster, ∼34, in both extremities as our simulated data involved 11,454 genes from 337 pathways. For each of the 48 clustering results, we evaluated their performance based on the BHI, V-measure, and ARI. The results are summarized in Fig. 2.

Although V-measure showed a strong correlation with the average number of genes in a cluster, BHI and ARI measures did not render their best performances at 70 average genes per cluster. This was more plausible as the average number of genes in the pathways used to generate the simulated data was ∼34. Overall, hierarchical, k-means, and EMMIXgene turned out to be the three top performers when different metrics and average number of genes per cluster were considered, followed by clusternomics and Pam. We continued to experiment with our atlas generating algorithm using the top three clustering methods.

### Cluster Merge

We clustered the simulated gene expression data with an expected number of genes per cluster of 25. For each cluster, we learned the GI network using BNP and noted the strength values among the genes within a cluster. The strength values were based on model averaging of 1000 bootstrap datasets. We then picked the representative genes for each cluster and generated the interaction network for the representative genes, again using the BNP approach with 1000 bootstrap datasets. The edges that have significant strength values were retained to determine the final network among the representative genes.

The interaction network among the representative genes was used as a map to guide the cluster merge step. If two representative genes were linked, we combined the genes in the two corresponding clusters and learned an interaction network for this union set using the BNP approach with 1000 bootstrap datasets. This resulted in a strength value between the genes that are in two different clusters. However, if a cluster ***C***’s representative node was linked to ***k*** other representative nodes, then the genes in this cluster ***C*** went through ***k*** cluster merge processes. This implied that there were ***k*** different strength values calculated for the genes that are in cluster ***C***, one for each of the cluster merge processes. We noted all of these ***k*** strength values for downstream analysis.

We analyzed merged clusters to see if they contained genes that belonged to the same pathway out of the 337 pathways used to construct the atlas. In 78% of the cases, the two merged clusters contained genes from the same pathway. This implied that using the interaction network of the representative genes to merge clusters enabled us to bring together the genes that belonged to the same pathway. These genes were separated in the clustering phase but now would be combined in the cluster merge phase to better capture the original pathway structure.

### Atlas Generation

We ran the complete workflow for our simulated dataset of 11,454 genes where we used hierarchical, k-means, and EMMIXgene clustering approaches for comparison purposes. Furthermore, the strength values among the genes in a cluster were taken to be either the strength value that is calculated when only the genes in the cluster were used to identify the GI network or the mean, median, minimum, maximum, one-step Tukey’s bi-weight average [81] of the ***k*** strength values obtained during the cluster merge step. Note that ***k*** represents the number of times a cluster goes through a merge process.

The true atlas used to generate the simulated data consisted of 17,777 edges. However, there are 65,591,331 possible edges between the nodes that make up the true interaction atlas. Therefore, there are far fewer “true edges” than there are “false edges.” Hence, for any accuracy assessment, in the context of identifying an edge as a “true” or “false” edge, the receiver’s operating curve (ROC) approach would not be appropriate. It has been suggested that for imbalanced datasets like this one, where the proportion of the true and false class labels are disproportionate, AUC of the precision-recall curve (AUC of PRC) is a better metric than the AUC of ROC [82].

In Table 1, we list the AUC of PRC values for our proposed atlas generation workflow using the three alternative clustering methods and six different strength value calculations for the genes in a cluster. While the baseline (pure random) performance for the AUC of ROC measure is 0.5, the baseline value for the AUC of PRC measure is the ratio of the true class. In our case, the baseline AUC of PRC measure is ∼2.7 × 10^−4^. As seen in Table 1, the hierarchical clustering approach outperformed other clustering methods yielding the highest AUC of PRC regardless of the strength calculation method. Furthermore, using the mean of strength values for a pair of genes within a cluster has consistently resulted in the best AUC of PRC value. Therefore, in our final atlas generation model we adopted this best performing approach (hierarchical + mean). The proposed workflow can be accessed at http://otulab.unl.edu/atlas.

**Table 1.** Area under the curve of precision-recall curve (AUC of PRC) (×10^−4^) values for the atlases generated using the proposed approach based on k-mean, hierarchical, or EMMIXgene clustering algorithms

Clustering method Link strengths	k-means	Hierarchical	EMMIXgene
First cluster	3.8	5.0	3.0
Minimum	29.3	39.0	13.0
Maximum	24.6	34.6	11.9
Mean	29.9	**39.7**	13.1
Median	26.2	36.3	12.7
Tukey	26.0	36.0	12.5

Strength values for the genes in a cluster were calculated either based on the strength value when only the genes in the cluster are used for network generation (First cluster) or the minimum, maximum, mean, median, and Tukey’s bi-weight average of the strength values obtained during the cluster merge process. For a pair of genes within a cluster, there were as many strength values as the number of times the cluster has gone through a merge process with another cluster. Best performing combination is highlighted with boldface and shaded background.

In order to compare our proposed workflow with other approaches, we used two main metrics that are used to generate large-scale interaction networks: correlation and average mutual information. For the proposed approach, we also tried the “perfect clustering” case where the 11,454 genes that make up the atlas were clustered into the 337 pathways that the genes came from. The results, summarized in Table 2, show that the proposed method outperforms conventional metrics in identifying the large interaction atlas.

**Table 2.** Area under the curve of precision-recall curve (AUC of PRC) values with 95% confidence interval for the atlases generated using the correlation and average mutual information (AMI) metrics compared with the proposed approach based on hierarchical clustering and perfect clustering of expression data

	Correlation	Hierarchical (proposed)	AMI	Perfect clustering (proposed)
AUC of PRC (×10^−4^)	7.9 [7.6–8.2]	39.7 [37.8–41.6]	2.4 [2.2–2.6]	5116 [4895–5337]

It is of note that the proposed algorithm with perfect clustering results in an AUC of PRC value that is about 130 times better than hierarchical clustering used in the proposed workflow. Although the proposed approach outperforms existing methods even with hierarchical clustering, there is still room for improvement in the clustering phase. The better the clustering results approximate the underlying biological organization, the more the generated atlas becomes similar to the true interactome.

### Clustering Effect on Learning

To understand the effect of clustering on learning, we generated 10 subnetworks from our large, true interaction atlas obtained from KEGG, each containing approximately 500 nodes. The subnetwork size was chosen such that it is not too large for the BNP approach (no clustering) to handle; and it is also large enough to justify the atlas approach (clustering) for network learning. We summarize the results in Table 3.

**Table 3.** Subnetwork statistics and the area under the curve of precision-recall curve (AUC of PRC) values for the learned networks using the Bayesian network prior (BNP) and atlas approaches

Subnetwork	No. of nodes	No. of edges	No. of pathways involved	AUC of PRC (×10^−3^)		
				BNP	Atlas	Baseline
1	528	879	11	129.6	112.8	6.3
2	542	840	13	109.8	95.5	5.7
3	496	913	13	113.9	102.5	7.4
4	514	911	8	112.2	96.5	6.9
5	537	850	11	74	59.9	5.9
6	526	897	13	124.8	109.8	6.5
7	494	837	11	124.2	105.6	6.9
8	459	845	14	83.2	69.9	8.0
9	508	912	8	108.8	87.0	7.1
10	462	816	8	79.3	63.4	7.7
Average	507	870	11.0	106.0	90.3	6.8
St. dev.	29.05	36.57	2.31	20.06	19.45	0.90

Each subnetwork was chosen to have ∼500 nodes.

Our results indicate that both BNP and atlas approaches achieve AUC of PRC values well above the pure random performance (baseline). The effect of clustering represented by the atlas approach renders about an 85% reduction in the overall performance. Furthermore, the average number of edges in the networks learned by the atlas approach was about 10% more than those learned by the BNP approach. This is potentially due to the cluster merge steps involved in the atlas approach that is likely to add more edges. Nevertheless, the performance obtained by our atlas approach significantly exceeds that of the baseline’s and is within a reasonable distance of BNP’s performance, which is promising, considering its ability to handle very large networks that are otherwise impossible to reconstruct without clustering.

### Application to Real Expression Data

We applied the proposed workflow to our previously established renal cell cancer (RCC) gene expression data that contained 23 normal and 32 clear-cell RCC samples [83]. We focused on 10 pathways, listed in Table 4, that have common genes and have been found to be associated with RCC using an experimental proteomic-based approach [84]. We had analyzed our expression dataset along with six other RCC datasets to infer active pathways using a Bayesian pathway analysis and these 10 pathways were found to be regulated [79].

**Table 4.** List of pathways used to generate the “mini-atlas” for testing the proposed workflow

KEGG pathway ID	Pathway name	No. of nodes	No. of edges
hsa00010	Glycolysis/Gluconeogenesis	28	78
hsa00020	Citrate cycle (TCA cycle)	16	40
hsa00030	Pentose phosphate pathway	20	61
hsa00061	Fatty acid biosynthesis	12	35
hsa00230	Purine metabolism	48	277
hsa00280	Valine, leucine, and isoleucine degradation	33	116
hsa00330	Arginine and proline metabolism	31	64
hsa00562	Inositol phosphate metabolism	27	75
hsa00620	Pyruvate metabolism	21	51
hsa00640	Propanoate metabolism	20	53

KEGG, Kyoto Encyclopedia of Genes and Genomes.

We constructed a “mini-atlas” with 213 genes and 892 edges by merging the 10 overlapping pathways listed in Table 4. Based on the expression of these 213 genes from our RCC dataset, we reconstructed the test atlas using the proposed workflow. Our approach generated nine clusters during the first iteration. After a network is learned for each cluster and clusters were merged using the representative GI map, we ended up with a network of 978 edges. The AUC of PRC for the learned network was 67.8 × 10^−3^, which was ∼17-fold better than the baseline AUC value of 4.0 × 10^−3^.

**Fig. 3.**
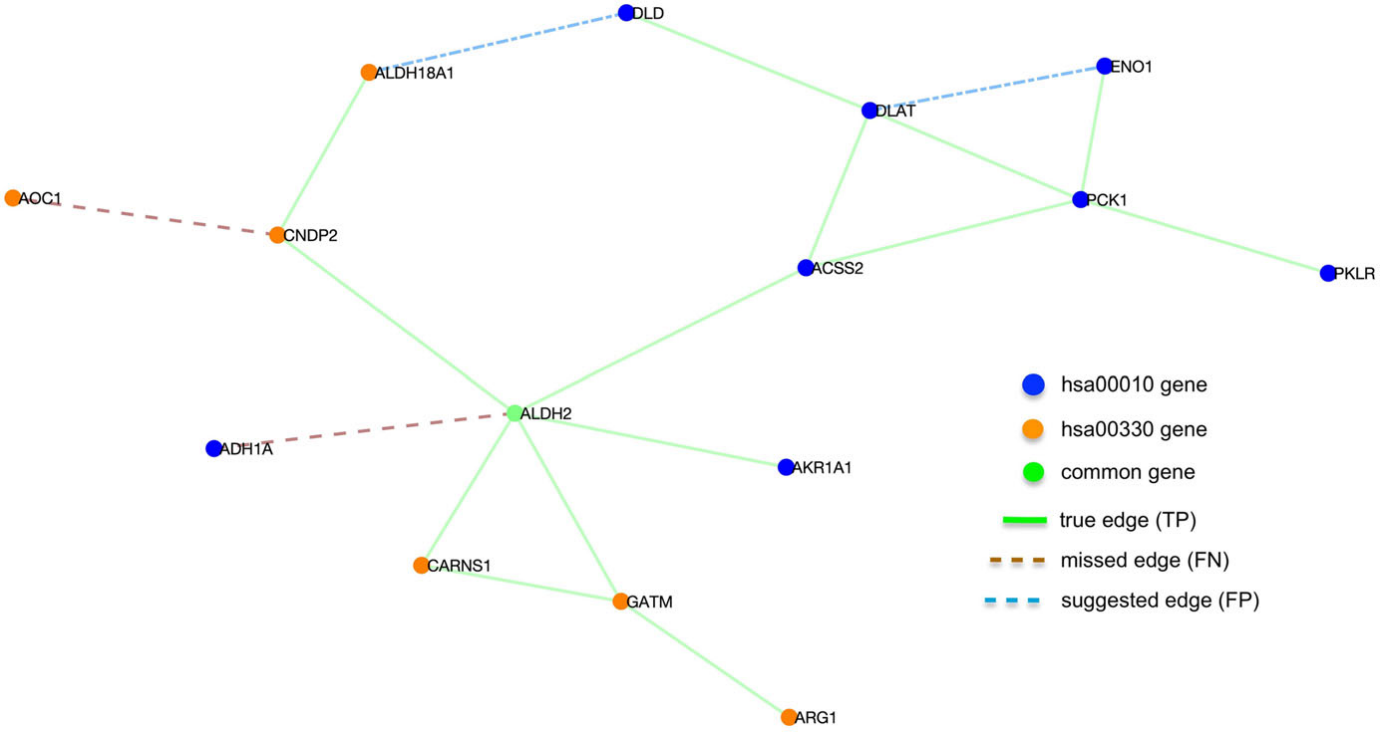
A subnetwork of the reconstructed test atlas that involves genes from the hsa00010 and hsa00330 Kyoto Encyclopedia of Genes and Genomes (KEGG) pathways. FN, false negative; FP, false positive; TP, true positive.

To demonstrate the utility of our approach, we focused on a subnetwork of the reconstructed atlas that contained genes from two networks: hsa00010 (Glycolysis / Gluconeogenesis) and hsa00330 (Arginine and proline metabolism). These two KEGG pathways have one gene, aldehyde dehydrogenase, in common. The subnetwork shown in Fig. 3 consists of 15 nodes and 18 edges. Our method correctly identified 14 edges that exist in these pathways (true positives), missed two edges (false negatives), and suggested two new edges (false positives) that do not exist in the true KEGG pathways.

The subnetwork shown in Fig. 3 demonstrates two important utilities of the proposed method. Firstly, the edge between the genes enolase 1 (ENO1) and dihydrolipoamide S-acetyltransferase (DLAT) do not exist in the KEGG pathway hsa00010 but is suggested by our method as a putative interaction. Indeed, there are studies that show significant association between these genes [85,86]. Therefore, the proposed method can suggest potential interactions that may not exist in current pathways but warrant further study for the given experimental data. Secondly, the edge between the genes dihydrolipoamide dehydrogenase (DLD) and aldehyde dehydrogenase 18 family member A1 (ALDH18A1) not only suggest a potential interaction that do not exist in the reference databases but also infers an edge that connects two pathways that are otherwise not connected. Like the ENO1–DLAT interaction, there exist studies that imply association between DLD and ALDH18A1 [87,88]. Hence, the proposed method has the potential to merge disconnected pathways and suggest interactions that do not exist in existing pathway databases but may be in play for the given experimental data.

## Discussion

Biological systems operate through a networked cascade of events and networks involving all direct and indirect interactions between genes and/or gene products describe the functional workflow of an organism’s biological machinery. When building the interactome of an organism, the biological databases that provide a vast amount of annotated data can be used in a systematic way. BN have become increasingly popular as they capture both linear and nonlinear interactions, handle stochastic events in a probabilistic framework accounting for noise, and focus on local interactions, which can be related to causal inference. However, interaction network learning algorithms are computationally very intense and feasible for only a limited number of nodes. Current methods have reached a bottleneck in terms of the size of the reconstructed network. In this paper, we proposed an algorithm to fix this bottleneck by developing a modular approach for reconstructing the entire interaction atlas for a complex organism. We demonstrated the effectiveness of our approach by constructing the interaction atlas for humans.

Our goal was to provide a divide-and-conquer approach that first identified groups of molecules that showed dependency based on experimental data. Within each group, or cluster, the corresponding interaction network is learned using external knowledge via the BNP framework. Each network is summarized using one representative node, all of which are used to build a meta-network representing the interactions between the clusters. The union of the nodes in interacting clusters underwent a second learning phase, and the ensemble of all the learned edges represented the final interaction atlas.

Using the simulated data that represented all the human pathways in the KEGG database, we optimized the proposed method for the clustering approach, cluster size, representative node selection, and cluster merge process. Our optimized parameter selection outperformed existing large-scale interactome generating metrics using the AUC of PRC measure. Our results suggested that the accuracy in the clustering phase of the proposed method dramatically impacted the reliability of the reconstructed interaction atlas. In the process of developing the atlas generation workflow, we also updated the BNP method and both the BNP approach, which can be used to reconstruct small interaction networks (with no clustering), and the atlas generation method described in this paper can be found at http://otulab.unl.edu/BNP and http://otulab.unl.edu/atlas, respectively.

Our current workflow has potential limitations. We can only operate on human transcriptomics and proteomics data for now. To apply the current approach to other organisms, we need to build the corresponding knowledge base that collects interaction information for those organisms based on external databases. We also cannot extend the current approach to other omics, such as metabolomics, or to a multiomics approach where an interaction network that involves different omic types is constructed. However, this can be possible when such a knowledge base, which lists interaction information between different omics, is established. Our current implementation does not identify network characteristics and motifs that result from the identified atlas. We hope to address these issues in our future work. Furthermore, despite bringing the ability to reconstruct very large interaction networks, clustering diminishes the performance of network learning by about 10–15% (Table 3). Despite our attempts to optimize each algorithmic step shown in Fig. 1, our approach still suffers methodological weaknesses. Primarily, there is room for improvement in hyperparameter selection in the employed clustering approaches as opposed to using the default values. Additionally, the network learning approaches, even though proven to be robust for gene expression data, are not able to produce faithful representations of the entire dependency structure but rather a subset of it.

The ability to generate large interaction atlases provides the means to understand the global characteristics and distant influences in the interactome. Most of the existing methods for interaction network generation focus on local modules at the pathway level, which does not provide the overall cause-and-effect mechanisms. Identifying interaction atlases can also be used to understand the characteristics of the interactome from a network science perspective. Coupled with biological interpretation of the interactions, this provides a tool to perform comparative analyses of disease mechanisms with a potential to lead to biomarker discoveries and/or putative therapeutic approaches.

## Data Availability

The software and data used in this publication are available at http://otulab.unl.edu/atlas.

## Supplementary material

For supplementary material accompanying this paper visit https://doi.org/10.1017/cts.2022.18. click here to view supplementary material
